# Contribution of remote Pacinian corpuscles to flutter-range frequency discrimination in humans

**DOI:** 10.1038/s41598-024-79693-5

**Published:** 2024-11-14

**Authors:** Saad S. Nagi, Sarah McIntyre, Kevin K. W. Ng, David A. Mahns, Ingvars Birznieks, Richard M. Vickery

**Affiliations:** 1 Center for Social and Affective Neuroscience, Linköping University, Linköping, Sweden; 2 School of Medicine, Western Sydney University, Sydney, Australia; 3 School of Biomedical Sciences, UNSW Sydney, Sydney, Australia; 4 Neuroscience Research Australia, Sydney, Australia; 5 Bionics and Bio-robotics, Tyree Foundation Institute of Health Engineering, UNSW Sydney, Sydney, Australia

**Keywords:** Touch receptors, Sensory processing, Neurophysiology

## Abstract

Among the various classes of fast-adapting (FA) tactile afferents found in hairy and glabrous skin, FA2 afferents, associated with Pacinian corpuscles (PC), preferentially signal high-frequency sinusoidal events corresponding with vibration percepts, in contrast to other classes associated with lower frequency flutter percepts. The FA2-PC complex is also uniquely sensitive to distant sources of vibration mechanically transmitted through anatomical structures. In the present study, we used a pulsatile waveform to assess the contribution of FA2 afferents to the perception of flutter-range frequency stimuli (~ 20 Hz) in combination with two methods to abolish local FA inputs and force a dependence on FA2 via transmission from adjacent structures. Firstly, we examined frequency discrimination and perception of vibration applied to the hairy skin overlying the ulnar styloid before and during the blockade of intradermal receptors by local anaesthesia. Secondly, we tested frequency discrimination on the digital glabrous skin before and during the blockade of myelinated fibres by ulnar nerve compression. Despite reliance on vibration transmission to activate remote PCs, we found that flutter-range frequency discrimination was unimpeded across both skin types. Comparisons with stimuli applied to the contralateral side also indicated that perceived frequency was unaffected. This confirms that flutter-range frequency perception can be encoded by the FA2-PC system. Our results demonstrate that input from receptors specialised for low-frequency signalling is not mandatory for flutter-range frequency perception. This explains how the constancy of frequency perception might be achieved across different skin regions, irrespective of the afferent type activated for transmitting these signals.

## Introduction

Vibratory deformations occur in the skin while undertaking routine tasks, such as exploring the textural properties of a surface or manipulating objects. These vibrations occur not only at the point of contact, activating local mechanoreceptors, but also transmit across large areas of the skin^1,2^, propagating from the fingers to the whole hand^3,4^ or even up the forearm and beyond^5^, detected by mechanoreceptors distant from the contact site. While many perceptual studies have focused on the vibrations produced at the point of contact^6,7^, the importance of remote mechanoreceptors is revealed in medical conditions such as carpal tunnel syndrome and traumatic median nerve section. In such cases, patients can still perceive the roughness of textures despite damage to or loss of the nerve supplying the fingers, implicating the involvement of primary afferents not necessarily located in the hand^8^. In this study, we investigated the perceptual consequences of vibration transmitted to skin sites remote from the point of stimulation in a healthy population by using compression block and local anaesthesia to block local afferent responses.

The classical view of human vibrotactile perception is based on the preferential activation of different classes of primary afferents, known as the “channel theory of frequency perception”^9^. Low-frequency inputs (< 50 Hz) are thought to be transmitted by fast-adapting (FA) fibres associated with Meissner corpuscles (termed fast-adapting type I, FA1) in glabrous skin, and by hair follicle afferents (HFA) in hairy skin. At higher frequencies (50 Hz to 300 Hz), vibrotactile information is thought to be signalled by FA2 fibres, which are associated with Pacinian corpuscles (PCs) in both glabrous and hairy skin^10–13^. PCs are located in the subdermal layers of glabrous skin but are more remote in the hairy skin, such as in the vicinity of joints and interosseous membrane^14–16^. Another class of FA fibres, called field units, are abundantly found in human hairy skin^17,18^, but their frequency preference has not yet been characterised.

The channel theory has been supported by the results of intraneural microstimulation experiments involving electrical activation of single afferents. These studies provide a direct link between afferent activation and perception. Activation of a single FA1 afferent evokes sensations of tapping, flutter or vibration at frequencies below 100 Hz^19,20^, while activation of a single FA2 afferent evokes a ticklish sensation at frequencies between 50 and 100 Hz and vibratory sensations at higher frequencies^21^.

In addition to FA afferents, slowly-adapting (SA) fibres in both skin types respond to vibration^22^. However, they do not encode information about vibration frequency with the same high fidelity as FA afferents. Specifically, there is a large gap between the thresholds for vibrotactile detection and the entrainment of SA afferents by vibratory stimuli^23^. Additionally, at vibration frequencies above 20 Hz, SA afferents are less sensitive compared to FA1 afferents^10,11,24^. Together, this suggests that SA afferents play, at best, a secondary role in vibration frequency signalling, whether in glabrous or hairy skin.

Vibration stimuli are typically delivered by a punctate probe oscillating perpendicular to the skin, producing repeated skin indentations at a given frequency and amplitude. With a sinusoidal waveform, the temporal profile of individual indentations changes with frequency^25^, such that higher frequencies have greater acceleration and velocity. Primary afferents are sensitive to these indentation properties, meaning the reported frequency tuning for different afferent classes is often confounded by the acceleration and velocity of the indentations when sinusoidal stimulation is used.

Furthermore, natural touch events are complex, composed of multiple frequency components as revealed by spectral analysis^26,27^. Analogous to pure tone signals in auditory research, sinusoidal vibratory stimuli have been extensively used in the tactile research reviewed above to isolate frequency components, and to investigate the functional role of mechanoreceptors in the skin and their associated primary afferents. However, some studies have shown that moving beyond pure sinusoidal vibration produces results which cannot be explained by the channel theory, and suggest that the temporal pattern of afferent spikes generated by vibration may be more important than which afferent classes are most activated^28,29^.

Our group has previously developed pulsatile mechanical stimuli that break the confounding link between vibratory stimulus frequency and the afferent population recruited^30,31^. These pulsatile stimuli have a brief protraction time of ~ 2 ms, which is independent of the repetition rate (frequency), and generate a single spike per pulse in each activated afferent, as confirmed during microneurography recordings^32,33^. This ensures that pulsatile stimulation recruits a fixed population of afferents depending on the amplitude, without being affected by frequency.

Using these pulsatile stimuli in glabrous skin, our earlier work challenged the prevailing view that vibratory frequency perception is strictly determined by the class of FA afferents activated. We demonstrated that low-amplitude (3 μm) mechanical pulses in the flutter range (20–40 Hz) can selectively recruit FA2 afferents and produce a low-frequency percept of vibration. The resulting percept has similar perceived frequency attributes to that produced by sinusoidal stimuli of corresponding frequencies, which are traditionally attributed to FA1 afferents^29^.

Since pulsatile stimuli can activate FA2 afferents at flutter-range frequencies, we leveraged this in the present study to investigate the contribution of remote skin sites to vibration frequency perception. In particular, we investigated frequency perception of flutter-range vibrations propagated to skin sites remote from the point of contact, likely signalled by FA2 afferents. Under ordinary circumstances, afferent responses at the point of contact will dominate perception. Here we used two approaches, nerve block and local anaesthesia, to knock out local inputs, while preserving the recruitment of remote FA2 inputs using pulsatile stimuli. We investigated both hairy and glabrous skin because FA2 afferents and PCs are found in both types of skin, and because remote transmission occurs in and across both skin types. Additionally, it is possible that any contribution of local inputs to vibration perception is different for these two skin types because the make-up of local skin afferents and end-organs is different in glabrous compared to hairy skin. We predicted that when local touch sensation is abolished by either anaesthetic injection or compression block, vibration discrimination performance would persist as a result of vibration transmission to FA2 afferents outside the affected area.

## Methods

We conducted three experiments with a total of 13 healthy participants (aged 20–45 years, 2 females). In Experiment 1, which included 12 participants, we tested the subjective capacity to discern between different frequencies in the ‘flutter’ range on the hairy skin overlying the ulnar styloid process, prior to and following intradermal anaesthesia. Six of these participants were also asked to perform bilateral comparisons (across both hands) following anaesthesia in Experiment 2. Furthermore, we tested discrimination capacity before and after compression blocks on myelinated fibres in seven participants (Experiment 3). Participant code labels are consistent across experiments, so for example, ‘P11’ in Experiment 1 is the same participant as P11 in Experiments 2 and 3. This study, conducted in 2013, was approved by the Human Research Ethics Committees of UNSW Sydney (HREC11074) and Western Sydney University (H9429). All experiments were performed in accordance with the Declaration of Helsinki. Written informed consent was obtained from all participants.

### Vibrotactile stimulation protocol

A circular probe with a rounded 5 mm diameter tip attached to a SignalForce GW-V4 shaker (Data Physics, UK) was positioned perpendicular to the skin surface. Stimulus generation and data acquisition were performed via a Power1401 mk II device using Spike2 (Cambridge Electronic Design, UK) and Matlab (Mathworks, USA) scripts. Voltage signals to the shaker were amplified with a SignalForce 30 W amplifier (Data Physics, UK). An OptoNCDT 2200 laser displacement sensor (Micro-Epsilon, Germany) was used to monitor probe movement.

For experiments involving hairy skin (Experiments 1 and 2), a resting indentation of 100 μm was chosen, while for glabrous skin (Experiment 3), a 50 μm indentation was chosen. These choices were in keeping with local tissue mechanics, to reduce skin compliance and maintain a stable contact between the probe and the skin surface. In order to ensure propagation of vibrations to remote sites, we did not use any aperture around the stimulation site that would dampen vibration. White noise was delivered through headphones to eliminate any auditory cues associated with the mechanical stimulator.

The stimuli employed in this study were pulsatile mechanical stimuli that have a sharp onset effective for activating PCs, but which can be delivered at low repetition rates. A detection task with these stimuli is independent of vibration frequency. This is because the pulse itself is a stereotyped waveform with a brief protraction time of ~ 2 ms. The frequency of stimulation (for example 20 Hz) is just the repetition rate of the delivery of these pulses. Subjects can detect a single one of these pulses, and the rate at which pulses are repeated does not impact that detection; so detection of the stimulus is not frequency dependent. Discrimination of frequency however requires the subject to utilise a train of these pulses, and these judgements do depend on the repetition rate of the pulses.

Low-frequency discriminative capacity was tested with pulsatile stimuli by applying a *standard* frequency of 20 Hz (40 μm), and a range of *comparison* vibration trains that were both higher (21, 22 and 24 Hz) and lower (16, 18 and 19 Hz) than the standard. The stimulus sequence was applied in a pseudorandom order, with the standard stimulus presented first in half of the trials and second in the other half. Each period of vibration lasted 1 s with an inter-stimulus interval of 0.5 s. By using a two-alternative forced choice paradigm, subjects were instructed to report whether the perceived frequency of the *first* or the *second* stimulus was higher by pressing one of two foot pedals in each trial. The next trial commenced 0.5 s after their response. Twenty trials were carried out for each combination of paired vibration trains.

### Experiments 1 and 2: local anaesthesia of intradermal receptors (hairy skin)

Participants were asked to sit with their right hand resting in a pronated (palm-posterior) position on a wooden hand model, affixed to the bench top and supported with vacuum pillows. Before starting the experiment, the hair on the skin overlying the ulnar styloid process was trimmed to prevent any cues associated with hair deflection that could interfere with verifying the anaesthetic block^34^. Local anaesthetic (0.2–0.4 ml of Xylocaine 2%) was injected at multiple sites into the hairy skin overlying the styloid process (the vibration site), thereby producing a transient blockade of all intradermal inputs from a ~ 10 mm diameter region. The blockade of intradermal receptors within the anaesthetised region was confirmed by the complete loss of sensation to light mechanical (von Frey: up to 2.0 g) and pinprick (using a sterile hypodermic needle) stimuli, which assess both large- and small-fibre function^34–36^, consistent with methods used in prior studies^12,37^. The loss of sensation was restricted to the site of anaesthesia, with the stimuli clearly perceptible when applied to a neighbouring, non-anaesthetised area of the skin.

In Experiment 1, after confirmation of the anaesthetic block, each combination of paired vibration trains was reapplied to the anaesthetised skin. To confirm that the intradermal block remained intact, testing with von Frey hair and pin prick was repeated, typically once every 5 trials, throughout frequency discrimination.

A separate test of perceived frequency was deemed valuable since it could theoretically be governed by separate processes to discrimination capacity. That is, eliminating local afferent cues close to the point of contact may bias the perceived frequency either up or down. For Experiment 2, we tested frequency perception with similar procedures as mentioned above. However, participants received the comparison frequencies on the left (unanaesthetised) ulnar styloid process and had to perform frequency judgements against the standard 20 Hz stimulus delivered to anaesthetised skin on the right styloid process.

### Experiment 3: conduction blockade of myelinated fibres by compression (glabrous skin)

Participants were asked to sit with their right hand in a supinated (palm-anterior) position on a hand model and supported with vacuum pillows. In order to minimise stimulus spread between the little finger (D5) and the other digits, the latter were secured affixed (taping/synthetic adhesive putty) to the hand model. In addition, a small piece of low-density foam was placed between D5 and the ring finger. Thereafter, the vibration probe was positioned perpendicular to the proximal palmar pad of D5.

In order to induce a compression block of the ulnar nerve, a small metal slab, with corners slightly cushioned with masking tape, was placed posteriorly, proximal to the medial epicondyle of humerus^34,38^. The testing of the frequency discriminative capacity of participants was timed to coincide with the preferential blockade of myelinated fibres, confirmed through standard detection tasks consistent with previous studies^34,37–40^. For these purposes, standard detection tests perform just as well as discrimination or perception rating tasks, while being less time-consuming, which is important given the dynamic nature of the block^41^. Compression blocks follow a large-to-small fibre gradient, with the large fibres blocked first, followed by the smaller fibres. To ensure, to the extent possible, that the Aβ fibres were indeed blocked, we waited until functional testing clearly indicated that the Aδ fibres were also affected before proceeding with the frequency discrimination task.

Participants were asked to verbally report whether they felt vibration at the point of stimulation – a test for Aβ function^35,36^, delivered at 20 Hz, 40 μm, using sinusoidal and pulsatile stimuli. They were also asked to draw where they felt the vibration on a hand drawing. Participants were also asked to verbally report whether they felt innocuous cooling – a test for Aδ function^35,36^, delivered using a ∼15 °C rod in contact with the skin for 5 s. During thermal testing, innocuous cold stimuli were alternated with innocuous warm stimuli in order to avoid any expectation effects. The progression of the compression block was verified by comparing the somatosensory sensibility in the ulnar territory with the skin overlying the index finger, which is innervated by the median nerve. Subjects were shielded from visual and auditory cues while performing these tests. Once it was confirmed that both vibration and cooling sensations were blocked, we proceeded with the frequency discrimination task.

### Data analysis

The proportion of responses for which the comparison stimulus was judged as having a higher frequency than the standard 20 Hz stimulus was calculated for each comparison frequency. Logistic regression was applied to these data to produce a psychometric function for each subject in each condition. From the psychometric functions, we used the slope parameter as the primary outcome measure indicating discrimination capacity. For the bilateral experiment (Experiment 2), the point of subjective equality (PSE) was calculated as the frequency corresponding to 50% judged as the higher frequency. The PSE is a measure of the perceived frequency of the standard 20 Hz vibration stimulus applied to anaesthetised skin.

In Experiments 1 and 3, we tested our predictions that when using either local anaesthetic or compression block to abolish touch sensation in a target area of skin, that vibration discrimination performance is preserved due to vibration transmission to receptors outside the affected area. Using the “ratio-of-scales heuristic”^42^ we scaled the post-anaesthetic (or post-compression block) slope parameter to the pre-anaesthetic (or pre-compression block) slope, with 0 indicating chance level discrimination and 1 indicating discrimination equal to the pre-anaesthetic performance. We hypothesised that the scaled slope of the psychometric function is significantly greater than 0 (Experiments 1 and 3). We performed one sample t-tests against a null value of 0 which is the expected value for subjects who are completely unable to discriminate frequency. This null hypothesis is predicted if frequency discrimination depends solely on the abolished local afferent responses. We also report the Weber fractions for both pre- and post-anaesthetic (Experiment 1) and pre- and post-compression block (Experiment 3). We did not use the Weber fraction for statistical analysis since it is undefined when the slope is 0.

Additionally, we performed Bayesian analysis, allowing us to compare two competing models. We used a point distribution at scaled slope = 0 as the null prior to represent the case in which frequency discrimination depends entirely on local touch inputs. We used a point distribution at scaled slope = 1 as the alternative prior to represent the case in which frequency discrimination is wholly preserved due to remote vibration transmission (a “competing-point hypothesis”^43^).

In Experiment 2, we tested whether the loss of local inputs due to anaesthetic led to any bias in perceived frequency. We performed a paired samples t-test to evaluate whether the post-anaesthetic PSEs were significantly different to pre-anaesthetic PSEs. We also used Bayesian analysis with a point distribution at PSE difference = 0 as the null prior to represent no difference. For the alternative prior we used a Cauchy distribution centred at PSE difference = 0 to represent a conservative expectation that smaller differences are more likely than large differences. A scale factor of 10 was used to ensure the distribution covers a theoretically plausible range of values, and a lower limit of − 20 was imposed to reflect the fact that perceived frequency (PSE) cannot be less than 0.

## Results

### Experiment 1: frequency discrimination after local anaesthesia of intradermal receptors

**Fig. 1:**
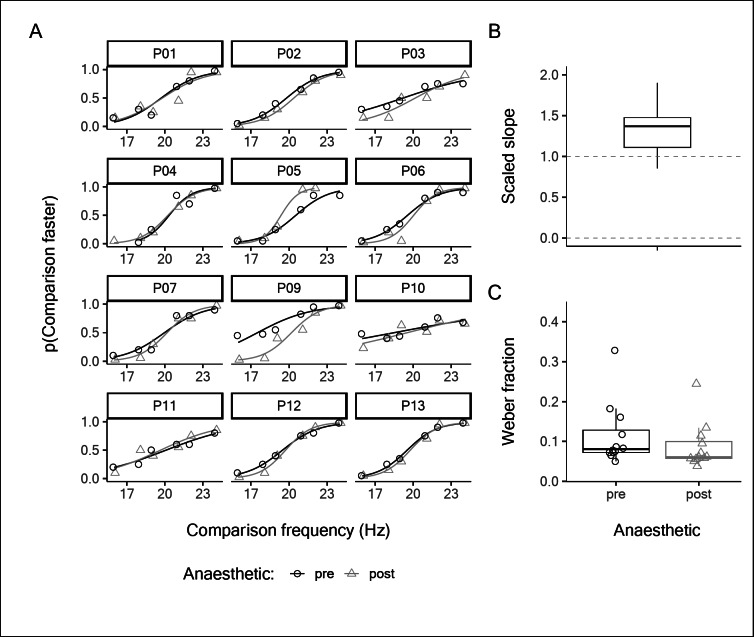
**A**: Individual psychometric functions for vibration frequency judgments on the ulnar styloid process, before and after anaesthetic was applied. **B**: The individual post-anaesthetic slope values scaled to the pre-anaesthetic slope values. Two predictions are indicated with dashed lines, 0 for abolished discrimination capacity, 1 for fully preserved discrimination capacity. **C**: Individual Weber fractions pre- and post-anaesthetic. The boxplots indicate the median, and first and third quartiles.

Participants at baseline were able to reliably discriminate the comparison frequencies from the standard frequency of 20 Hz, as shown by the black psychometric curves in Fig. 1A. Subsequently, subjects received multiple injections of local anaesthetic around the ulnar styloid process. Within the anaesthetised area of skin, all subjects were insensitive to light mechanical and pinprick stimuli. Subjects also reported a qualitative change in sensation, such that the sense of local pressure at the vibration site was abolished, while the sense of diffuse vibration persisted. Despite this, participants appeared to have preserved frequency discrimination, visible in the psychometric curves for each subject after anaesthesia shown by the grey curves in Fig. 1A.

The scaled slope parameter (Fig. 1B) was significantly greater than 0 (*mean* = 1.34, *95% CI* = [1.12, 1.56], *t*_*11*_ = 13.5, *p* = 3.40 × 10^− 8^, *d* = 3.90, 95% *CI* = [2.19, 5.60]) indicating that vibration discrimination capacity was not abolished despite the elimination of local touch receptors by the anaesthetic. The Bayesian analysis provided strong evidence for complete preservation of discrimination capacity over its abolition (BF = 376,785). Before anaesthetic, the median Weber fraction was 0.08 (range: 0.05, 0.33), and after anaesthetic was applied, the median Weber fraction was 0.06 (range: 0.04, 0.24; Fig. 1C). Surprisingly, the results actually suggest an improvement in discrimination capacity may have occurred with anaesthetic, which is most easily observed for P05 and P09 (Fig. 1A).

### Experiment 2: bilateral perceived frequency after local anaesthesia

**Fig. 2:**
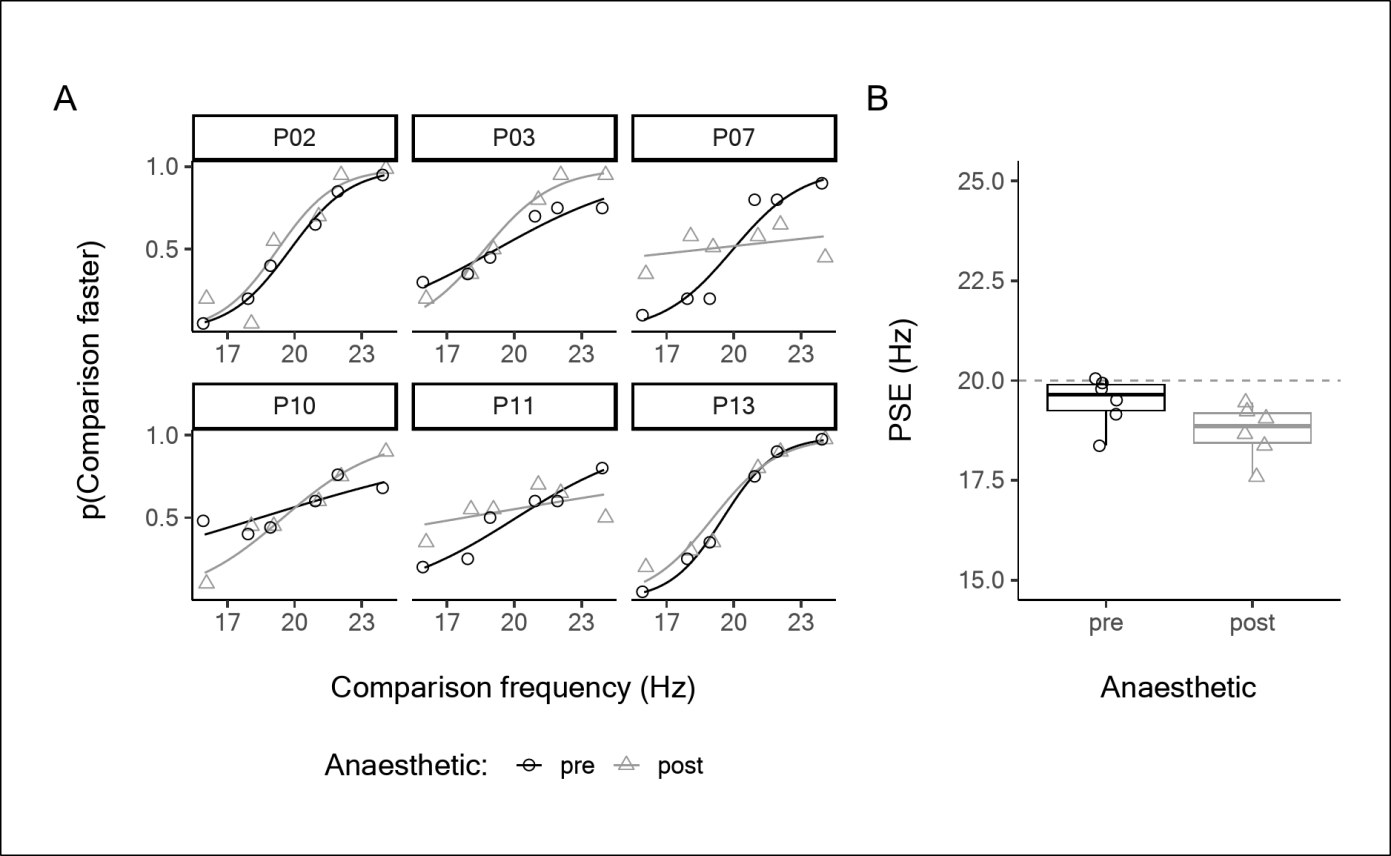
Bilateral comparison of frequency applied to the styloid processes, pre- and post-anaesthesia of the skin overlying the right styloid. **A**: Individual psychometric functions. **B**: Individual perceived frequencies, indicated by point of subjective equality (PSE). Horizontal line at expected stimulus frequency of 20 Hz. The boxplots indicate the median, and first and third quartiles.

The results of Experiment 1 show that frequency discrimination capacity was largely unaffected by intradermal local anaesthesia. However, it is still possible that the intervention could have influenced the perceived frequency of vibration stimuli. When vibration is applied to anaesthetised skin we are forced to rely on remotely transmitted vibrations. We tested whether this resulted in a bias to perceive the stimulus as either higher or lower frequency compared to unanaesthetised skin which received both local and remote transmitted signals. To test for this possibility, participants compared the frequency of stimuli applied to the left and right styloid processes. The PSE for the right stimulus (where anaesthesia had been applied) was determined by varying the frequency presented to the left styloid. The bilaterally determined PSEs were a good match to the actual stimulus frequency of 20 Hz delivered to the right styloid (Fig. 2), both before (mean PSE = 19.5 Hz, SD = 0.6) and after anaesthetic (mean PSE = 18.7 Hz, SD = 0.7). The mean difference was not significantly greater than 0 (*mean diff* = 0.7 Hz, *95% CI* = [− 0.5, 2.0], *t*_*5*_ = 1.5, *p* = 0.190, *d* = 0.62, 95% *CI* = [− 0.29, 1.48]) and the Bayesian analysis provided moderate evidence in support of the null model that perceived frequency is not biased by the elimination of local cues (BF = 0.148).

### Experiment 3: frequency discrimination after conduction blockade of myelinated fibres by compression

We also conducted an experiment stimulating the palmar pad of the little finger (D5) prior to and during compression block of the ulnar nerve. Seven subjects participated in this experiment, and a successful nerve block was achieved in five of them. In the other two subjects, a clear block of the myelinated fibres could not be achieved as determined by the somatosensory tests; this attrition rate is common in this type of procedure^34,44^. In all subjects, the stimulation site was the proximal phalanx of D5, except for P11, in whom it was the middle phalanx of D5. Subjects with a successful nerve block were unable to detect the 20 Hz, 40 μm sinusoidal stimulus delivered to the base of D5. However, they could detect the 20 Hz, 40 μm pulsatile stimuli delivered to the same site, but reported that the sensation originated only in the neighbouring (median nerve) territory and not at the stimulation site (no focal sense). The detection of innocuous cold stimuli was abolished.

**Fig. 3:**
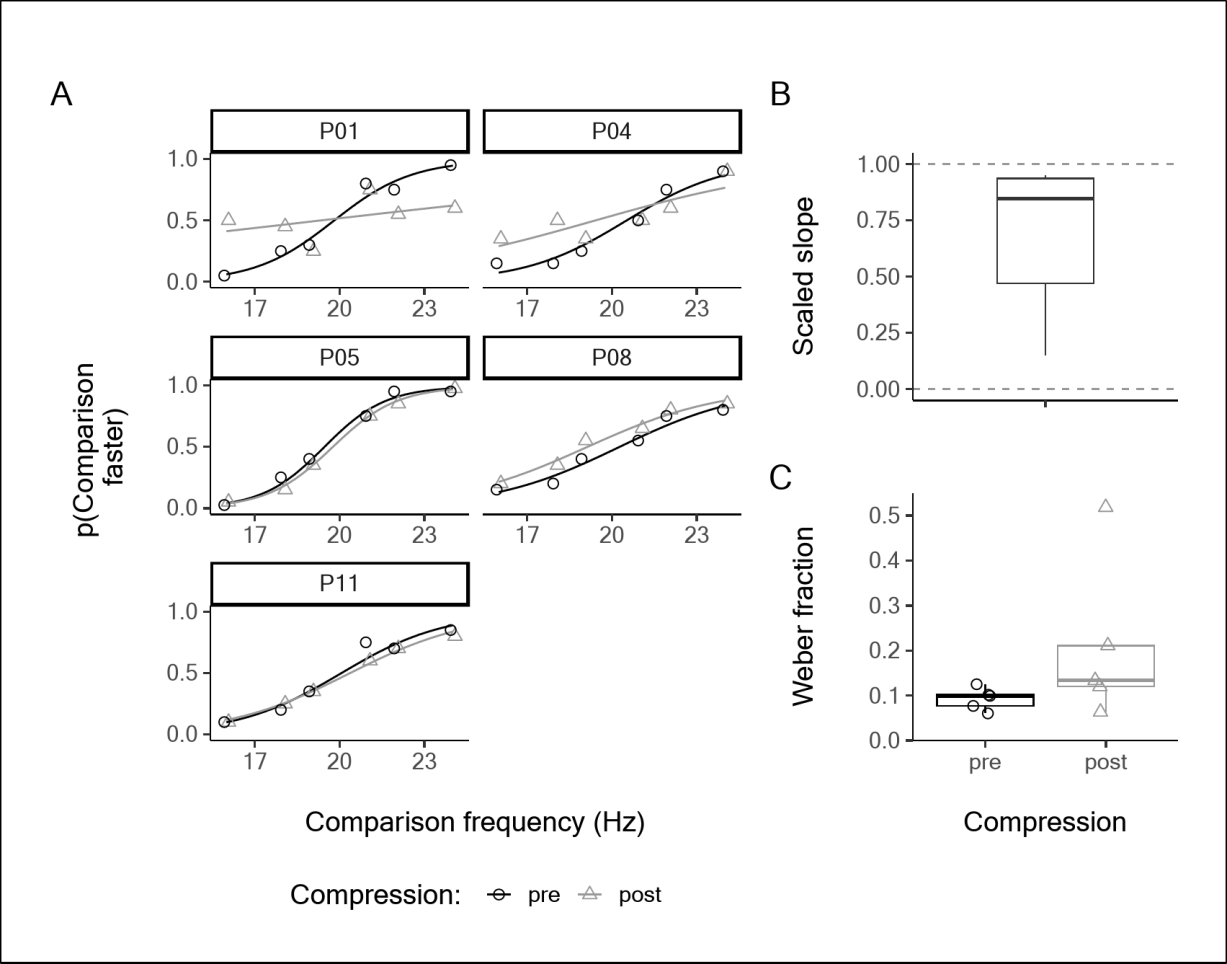
**A**: Individual psychometric functions for vibration applied to glabrous skin on the fifth digit pre- and post-compression block of the ulnar nerve. **B**: The individual post-anaesthetic slope values scaled to the pre-anaesthetic slope values. Two predictions are indicated with dashed lines, 0 for abolished discrimination capacity, 1 for fully preserved discrimination capacity. **C**: Individual Weber fractions pre- and post-anaesthetic. The boxplots indicate the median, and first and third quartiles.

Participants were able to reliably discriminate the comparison frequencies from the standard frequency of 20 Hz, both pre- and post-compression block, visible in the psychometric curves for each subject in Fig. 3A. The scaled slope parameter (Fig. 3B) was significantly greater than 0 (*mean* = 0.67, *95% CI* = [0.23, 1.11], *t*_*4*_ = 4.3, *p* = 0.013, *d* = 1.90, 95% *CI* = [0.34, 3.42]) indicating that vibration discrimination capacity was not abolished despite the elimination of local touch receptors by the compression block. The Bayesian analysis provided moderate evidence for complete preservation of discrimination capacity over its abolition (BF = 11.4). Before compression block, the median Weber fraction was 0.10 (range: 0.06, 0.13). During the compression block, the median Weber fraction was 0.13 (range: 0.06, 0.52; Fig. 3C). Although discrimination capacity was not abolished, the results suggest there may have been some reduction due to compression block. This seems to be driven primarily by P01 and P04, who had shallower slopes for their psychometric functions during the block (Fig. 3A).

## Discussion

It has been previously shown that intradermal anaesthesia of the hairy skin overlying the brachioradialis muscle significantly impairs sinusoidal frequency discrimination in the flutter range^12^. In that study, a 20 Hz, 500 μm sinusoidal stimulus was reported as imperceptible, and the Weber fraction for 50 Hz frequency discrimination was approximately 1, compared with ~ 0.35 in the unanaesthetised condition. This residual poor discriminative ability was suggested to be due to the spread of the stimulus to deeply located PCs. An implication of this suggestion is that frequency processing of PC/FA2 inputs may be poor for low frequencies of activation. The present study set out to address this question more directly by combining local inactivation of FA receptors with the use of pulsatile stimuli that have a sharp onset effective for activating PCs, but which can be delivered at low repetition rates. This enabled us to directly observe the effect of an FA2-only signal remote to the stimulus site, that was entrained to low vibration frequencies traditionally associated with FA1 inputs^29^.

The intradermal injection of local anaesthetic is a well-established technique to inactivate afferents located in the skin^12,37^. As PC receptors are not located within the hairy skin but in the vicinity of joints and bone, the skin overlying the ulnar styloid process was chosen for our stimulus site, so that the distance for stimulus spread to the remote, non-anaesthetised PC receptors was smaller. By stimulating the hairy skin over the ulnar styloid process with a low-amplitude stimulus (40 μm) in the centre of the ~ 80 mm^2^ zone of anaesthesia, we believe the local FA afferents would be inactivated by the anaesthetic, while FA receptors in the adjacent unanaesthetised skin would receive a stimulus below their threshold amplitude. This is on the basis that the detection threshold on forearm hairy skin is > 100 μm at 50 Hz, and 50 μm at 200 Hz^12^, with the minimum threshold around 200–300 Hz^45^. Our pulsatile stimulus has some energy at a frequency of around 500 Hz, but the threshold rises beyond 300 Hz, and so the detection thresholds are unlikely to be much lower than those quoted.

Studies have shown a notable frequency- and location-dependent reduction in the amplitude of human skin vibrations measured at a site distant to the stimulus probe^4,46^. But these were conducted in glabrous skin and so cannot be directly extrapolated to hairy skin. However, given the reported thresholds for hairy skin, and the reasonable assumption of significant attenuation with distance, we believe that our pulsatile stimuli were not activating HFA receptors. In glabrous skin, we used a conduction block of the ulnar nerve to prevent A-fibre inputs from its innervation territory, including the whole of D5. Stimulation was delivered at the base of D5, which gave a minimum distance for vibration to travel and reach a zone innervated by the median nerve of at least 15 mm. The vibration amplitude of the skin at this distance should be less than 4 μm^46^, which is well below the threshold for FA1 afferents^47^. Therefore, during both forms of nerve block, we believe the stimulus was primarily activating FA2 afferents located deep beneath or remote to the stimulation site. Indeed, animal studies have demonstrated FA2 activation by remote vibrotactile stimuli. Blocking nerve transmission from the mouse forepaw by injecting local anaesthetic into the wrist caused a reduction but not abolition in cortical evoked responses, which suggests that information from mechanoreceptors located in the paw and remote receptors in the forelimb converge to drive common cortical neurons^48^.

With either method of inactivating all FA inputs from the skin stimulation site and leaving only the deep and/or remote FA2 inputs intact, subjects were still able to perform frequency discrimination in the flutter range (Figs. 1 and 3). We even found a small improvement in average performance with the anaesthetic block (Fig. 1), although we believe this to be the result of variability and have no physiological significance, given that Experiment 3 showed no equivalent increase (Fig. 3), and that the Weber fraction remained close to the value reported in the literature^49^. With the ulnar nerve block (Fig. 3), two subjects had lower Weber fractions, but the other subjects showed essentially unaltered discriminative ability, despite being reliant only on the spreading skin vibration to activate the remote FA2 afferents. Additionally, the frequency perceived by subjects faithfully matches that expected by the stimulus frequency, i.e. 20 Hz (Fig. 2).

A substantial portion of normal FA2 activation is due to the spreading of skin vibration to remote sites up to at least 64 mm distance from the site of stimulation^46^. Human psychophysical studies have shown that the capacity to detect and discriminate high-frequency sinusoidal vibrations (> 80 Hz) remained intact following anaesthesia and nerve lesions^12,50^. Even in normal conditions where the applied frequency is low (< 100 Hz), the population entrainment of neural activity in the FA2 afferents is very well preserved^46^. This population entrainment presumably provides the basis for the preserved frequency discriminative ability that we have observed.

Our current observation that intradermal anaesthesia does not impair frequency discrimination suggests that the signal coding for frequency in the flutter range may well be widely distributed across the population of activated fibres (associated with HFA and PC in the case of hairy skin), with possible redundancy such that receptors beneath the stimulation probe and those located more remotely are equally effective in producing a sense of frequency. Although in the central nervous system, recordings from dorsal column nuclei, thalamus, and somatosensory cortex originally suggested that there are two classes of rapidly adapting neurons operating in two bandwidths of low and high frequencies, more recent evidence shows clear convergent inputs from HFA/Meissner and PC sources onto single cortical neurons^24,51,52^. This convergence may provide a basis for how low-frequency information is relayed to the cortex via afferents associated with PC receptors, as indicated by the present study.

Moreover, these findings might be further evidence for a more generalised frequency processing mechanism that can operate on inputs originating from different skin regions, despite being innervated by different receptors or afferent types, for maintaining constancy of frequency perception^31^. A temporal coding mechanism based on the timing of inter-spike intervals, as opposed to afferent class recruited, has been previously proposed in studies involving both pulsatile mechanical stimuli^29,53^ – where low-frequency flutter perceptions could be evoked with FA2 activation – and electrical stimuli^54–56^; the latter demonstrated similar frequency coding mechanisms as mechanical studies, despite bypassing mechanoreceptors and activating all afferent classes non-selectively. Such a temporal coding scheme has also been recently suggested for auditory stimuli^57,58^.

In conclusion, combining blockade of local FA receptors with pulsatile stimuli allowed us to demonstrate that information about flutter-range stimuli can be effectively encoded by remote PC mechanoreceptors in both hairy and glabrous skin. That is, the perception of low-frequency vibration can be signalled by receptors not necessarily located in the immediate cutaneous/sub-cutaneous tissues underlying the stimulation site, underscoring the importance of the mechanical properties of these tissues and the exquisite sensitivity of PC receptors to remote vibratory disturbances. In contrast to earlier reports using sinusoidal vibration in hairy skin^12^, we have shown that these remote receptors not only preserve the ability to detect these vibratory stimuli but also effectively convey the information, ensuring that frequency discriminative ability is unimpaired by the loss of local HFA, field, or Meissner receptor input. Given that most natural stimuli activate all classes of tactile afferents, this shared processing is presumed to support the robust and stable processing of complex vibrotactile features.

## Data Availability

Data and analysis scripts associated with this study are available on Open Science Framework: https://osf.io/5wxaq/
